# Effective Connectivity Predicts Surgical Outcomes in Temporal Lobe Epilepsy: A SEEG Study

**DOI:** 10.1111/cns.70563

**Published:** 2025-08-26

**Authors:** Xu Hu, Yuan Yao, Baotian Zhao, Xiu Wang, Zilin Li, Wenhan Hu, Chao Zhang, Kai Zhang

**Affiliations:** ^1^ Department of Neurosurgery Beijing TianTan Hospital, Capital Medical University Beijing China; ^2^ Department of Neurosurgery No. 904 Hospital of the PLA Joint Logistics Support Force Wuxi China; ^3^ Department of Neurosurgery Beijing Neurosurgical Institute, Capital Medical University Beijing China

**Keywords:** drug‐resistant epilepsy, effective connectivity, stereo‐electroencephalography, temporal lobe epilepsy

## Abstract

**Introduction:**

Temporal lobe epilepsy (TLE), the most common type of drug‐resistant epilepsy (DRE), has a postoperative seizure‐free rate of ~70%. Furthermore, precisely localizing the epileptogenic zone and determining the surgical resection area have been established as the key factors influencing surgical outcomes. Herein, we innovatively coupled the surgical resection area with characteristics of effective connectivity via intracranial electroencephalography (iEEG) to predict patients' surgical prognosis.

**Methods:**

This study involved 56 patients who underwent TLE surgery and were followed up for over 1 year. All patients underwent stereo‐electroencephalography (SEEG) electrode implantation and single‐pulse electrical stimulation (SPES) tests. After comparing patients' RMS value of N1/N2 (Z‐score standardized) from cortico‐cortical evoked potentials (CCEP) with different surgical outcomes, an interpretable machine learning (ML) model based on support vector machine (SVM) for predicting patients' surgical prognosis was constructed.

**Results:**

Patients with various surgical outcomes exhibited differences in effective connectivity. Furthermore, compared to the seizure‐free group (Engel I), patients in the nonseizure‐free group (Engel II‐IV) exhibited stronger connectivity between the seizure onset zone (SOZ) and regions outside the surgical resection area. The nonseizure‐free group also exhibited stronger connectivity between the surgical resection area and regions outside the resection area. Our prediction model demonstrated high‐accuracy performance, with accuracy and area under the curve (AUC) values of 0.800 and 0.893, respectively.

**Conclusions:**

This study confirmed the potential value of integrating the surgical resection area and effective connectivity characteristics in predicting patients' surgical outcomes; offering a novel approach that could be leveraged to precisely determine the surgical resection area and improve TLE patients' surgical prognosis.

## Graphical abstract

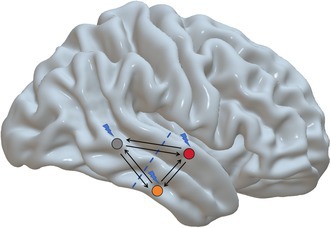

## 1 Introduction

Temporal lobe epilepsy (TLE), the most common type of focal epilepsy, accounts for ~66% of all cases [1]. Notably, ~71% of TLE patients are drug‐resistant [2], making most of them potential candidates for surgical interventions [3]. Anterior temporal lobectomy (ATL) is the standard neurosurgical procedure for TLE [4]. However, long‐term seizure freedom is achieved in only 60%–80% of patients following ATL [5, 6]. This phenomenon could be attributed to the complexity of the mechanisms underlying TLE networks and the intricate distribution of epileptogenic zones, making it difficult to accurately localize the epileptogenic region using conventional preoperative evaluation methods [7, 8, 9]. Recent advances in neuroimaging and electrophysiological techniques have enabled more comprehensive investigations of epilepsy networks. These methods aim to identify the true epileptogenic zone and critical network nodes, with the ultimate goal of improving surgical outcomes [10, 11].

Stereo‐electroencephalography (SEEG)‐based intracranial EEG (iEEG) plays a vital role in localizing the epileptogenic zone in patients with complex epileptic networks. Unlike scalp EEG, which captures surface cortical activity and is prone to signal attenuation and spatial blurring, SEEG directly records from deep brain structures with high spatial and temporal resolution. Moreover, SEEG provides an ideal platform for examining epilepsy networks. According to research, effective connectivity obtained through cortico‐cortical evoked potentials (CCEPs) could establish causal relationships across different brain regions, offering robust connectivity evidence [12, 13]. This is conceptually distinct from structural connectivity, which refers to physical white matter tracts (e.g., DTI), and functional connectivity, which captures statistical correlations in activity patterns (e.g., fMRI) without implying directionality or causality. CCEPs are recorded via single pulse electrical stimulation (SPES) to contact pairs of SEEG electrodes and observe the evoked potential in remaining electrodes, highlighting their potential utility in exploring pathological and functional large‐scale brain networks in the human brain. Multiple previous studies on epilepsy have explored the potential of using CCEP to localize epileptogenic zones and map epilepsy networks [12]. A high amplitude of evoked potentials following stimulation could reflect an increased excitability of the cortex, which may be related to tissue epileptogenicity [14, 15]. Furthermore, CCEP connectivity characteristics were reported to correlate with the propagation networks during the ictal stage [16].

Despite prior studies exploring the utility of CCEP in mapping epileptic networks or identifying SOZ [17, 18, 19], most have remained descriptive or limited to correlation analyses, lacking predictive frameworks that incorporate both connectivity measures and surgical resection extent, which limits the clinical translation of their findings. Moreover, these studies often fail to leverage machine learning approaches to quantitatively link connectivity features with long‐term seizure outcomes. Herein, 56 TLE patients were recruited to investigate effective connectivity relationships between the seizure onset region (SOZ) and areas both within and outside the surgical resection zones, as well as their utility in predicting TLE patients' surgical outcomes against the gold standard of seizure freedom for at least 1 year according to the Engel classification [20]. Unlike previous studies that either focused solely on CCEP network features or clinical outcomes, our approach is methodologically novel in that it explicitly integrates individualized CCEP‐based connectivity profiles with actual resection zone boundaries. This integrated framework enables a direct and interpretable model for predicting surgical prognosis and provides actionable insights for optimizing resection strategies in clinical practice.

## 2 Materials and Methods

### 2.1 Patient Selection

This study involved SEEG‐monitored drug‐resistant epilepsy (DRE) patients who underwent tailored ATL based on the monitoring results obtained between January 2021 and January 2023 at our epilepsy center. The inclusion criteria were as follows: (1) age ≥ 18 years; (2) diagnosis of unilateral TLE based on clinical semiology and noninvasive evaluation; (3) failure of at least two appropriately selected and dosed antiepileptic drugs; (4) clear seizure onset zone (SOZ) confirmed by SEEG; and (5) surgical resection performed according to SEEG findings, with postoperative follow‐up duration ≥ 12 months. Exclusion criteria included: (1) extratemporal or multifocal epilepsy; (2) severe psychiatric or neurodevelopmental comorbidities that could confound seizure outcomes; (3) presence of large structural lesions not confined to the temporal lobe on MRI; and (4) incomplete clinical or imaging records. All patients underwent SPES testing during the SEEG monitoring and followed up for more than 1 year. Before SEEG implantation, all patients completed a comprehensive set of presurgical evaluations. All patients' clinical data were carefully reviewed, including clinical history, seizure semiology, neurological examination, neuropsychological assessment, multimodal neuroimaging, and scalp‐EEG. Before resection surgery, patients' SEEG results and other clinical data were carefully reviewed by a multidisciplinary team, including neurologists, neurosurgeons, radiologists, and electrophysiologists, to develop a surgical plan and determine the extent of the resection. Two senior neurosurgeons (K.Z. and C.Z.) performed all resection surgeries. This study was approved by the institutional review board of Beijing Tiantan Hospital, Capital Medical University (KY2022‐016‐01), and written informed consent was obtained from all participants involved.

### 2.2 Imaging Data Acquisition

Before resection surgery, the following imaging data were collected for preoperative evaluation and SEEG implantation planning: (1) three‐dimensional magnetization prepared rapid acquisition gradient echo sequence (T1WI‐3D‐MPRAGE; repetition time [TR] = 2200 ms, echo time [TE] = 2.26 ms, slice thickness = 1 mm, and voxel size = 1 mm × 1 mm × 1 mm); (2) three‐dimensional fluid attenuated inversion recovery sequence (3D‐FLAIR; TR = 6500 ms, TE = 431 ms, slice thickness = 1 mm, and voxel size = 1 mm × 1 mm × 1 mm); (3) three‐dimensional time of flight magnetic resonance angiography (3D‐TOF‐MRA; TR = 20 ms, TE = 3.43 ms, slice thickness = 0.63 mm, and voxel size = 0.3 mm × 0.3 mm × 0.6 mm); (4) magnetic resonance venography (MRV; TR = 20 ms, TE = 5 ms, slice thickness = 2.5 mm, and voxel size = 1 mm × 1 mm × 2.5 mm); and (5) fluorine‐18 fluorodeoxyglucose positron emission tomography (18‐FDG PET). The patients were further subjected to thin‐slice computed tomography (CT) scans (0.625 mm) on the same day following SEEG implantation and resection surgery to confirm the presence of postoperative hemorrhage, electrode placement, and extent of surgical resection.

### 2.3 Electrode Localization

Individual space coordinates of electrode contacts (Sinovation, Beijing, China; diameter = 0.8 mm; 8–16 contacts, each 2 mm in length with 1.5 mm spacing) were determined using the SinoPlan software v2.0 (Sinovation, Beijing, China). To align with the bipolar reference scheme of SEEG signals, the midpoint between adjacent electrode pairs was determined and designated as each bipolar channel's position. Only contacts located within the gray matter were included in the analysis. First, a binary gray matter mask in individual space was produced by the SPM12 [21] segmentation procedure with a threshold of 0.1. A 3 × 3 × 3 voxel cube centered on the midpoint of two adjacent contacts was modeled to sample the surrounding voxels. The channel was labeled as gray matter if the cube contained ≥ 24 gray matter voxels, which was sufficiently strict to exclude irrelevant white matter channels. The segmentation results were visually inspected by two trained reviewers to ensure accuracy. Postresection CT was further co‐registered with post‐SEEG CT, and patients' binary resection masks were manually created using VOI tools in MRIcron based on postresection CT scan results. To ensure segmentation reliability, two experienced neurosurgeons independently performed the delineation for each patient. Inter‐rater agreement was assessed using the dice similarity coefficient, yielding a mean Dice score of 0.82 ± 0.08 across all patients, indicating good consistency between raters. Following that, contact pairs were further categorized as either in (IR) or outside (OR) the resection area using custom MATLAB scripts (MATLAB 2021a, MathWorks).

### 2.4 Single Pulse Electrical Stimulation (SPES)

**FIGURE 1:**
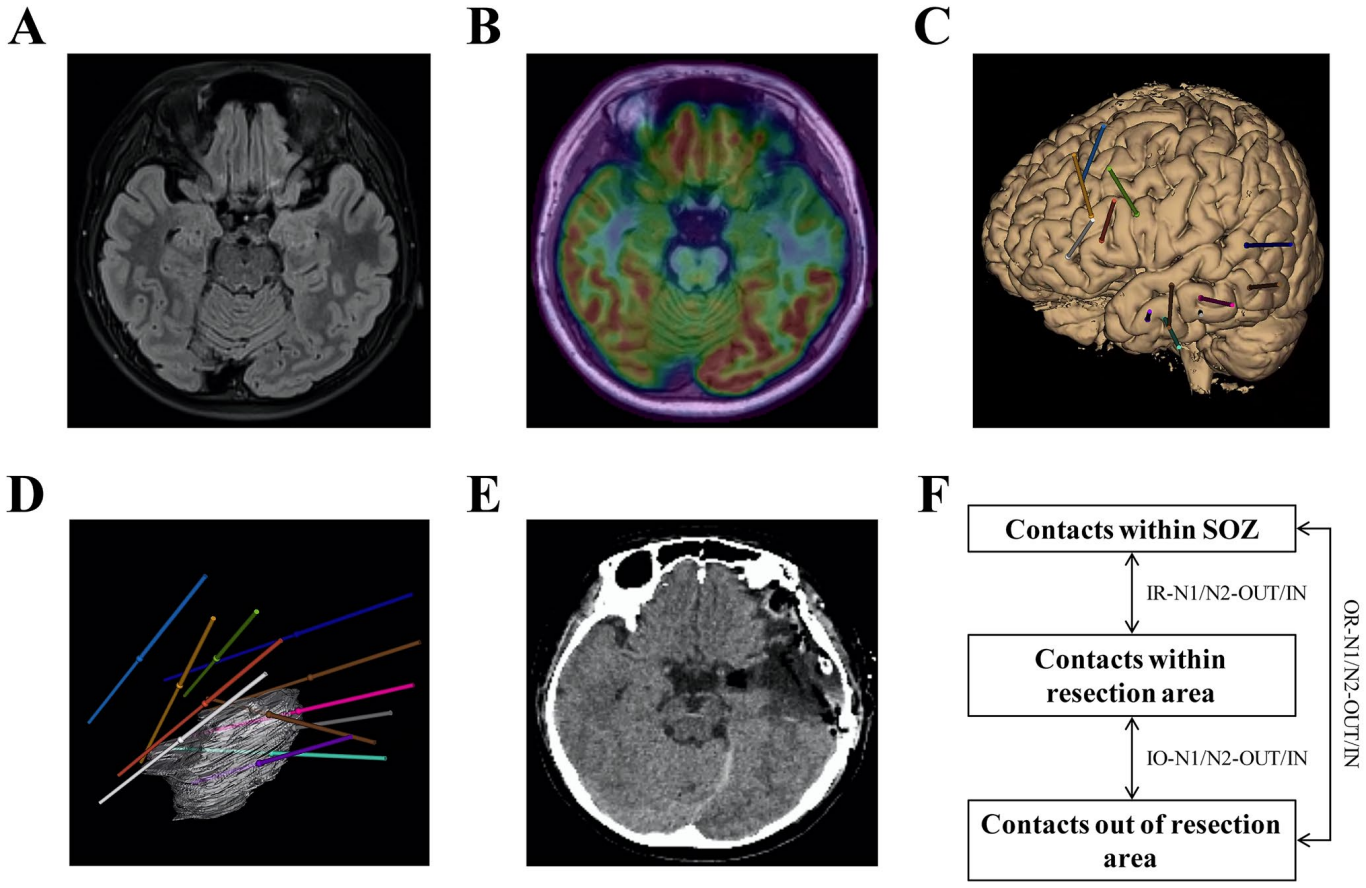
Study workflow: (A, B) Presurgery FLAIR and PET image of one case; (C) The case's SEEG electrode implantation diagram; (D) The case's thin‐cut CT image after resection surgery; (E) The resection region's ROI and trajectories of SEEG electrodes; and (F) CCEP parameters calculated per contact locations.

**FIGURE 2:**
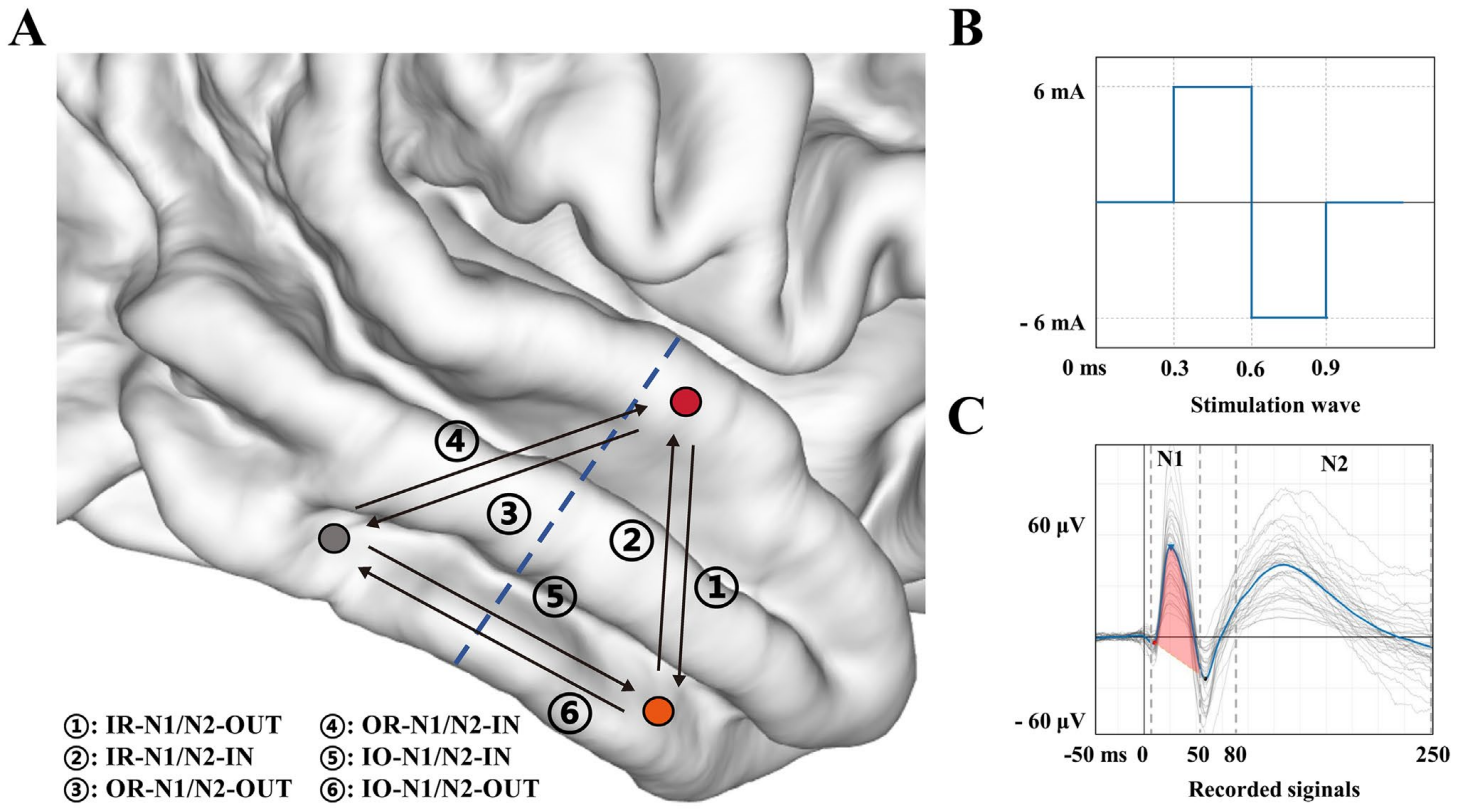
Illustration of CCEP calculation: (A) All CCEP parameters in analysis. Red dot: Contact in the SOZ; Gray dot: Contact out of the resection area; Orange dot: Contact within the resection area; Blue line: Resection boundary. (B) A single pulse stimulation wave; and (C) Signals in a recorded channel.

First, bipolar single pulses of stimulation (6 mA, biphasic, 300 μs phase duration) repeated 40× at 0.5 Hz were applied sequentially between two adjacent contacts of all electrodes. The stimulation parameters used in this study are consistent with established CCEP protocols. These parameters have been widely adopted in previous human SEEG studies and are considered safe, with no reported risk of afterdischarges, seizures, or tissue injury [12, 16]. Data recorded simultaneously from nonstimulation sites were then collected. Before analysis, the recorded signals were first preprocessed as follows: (1) Notch filtering of the line noise and its harmonics; (2) Referencing to the bipolar montage; (3) Bandpass filtering between 1 and 300 Hz; and (4) Downsampling to 1000 Hz. Following that, continuous EEG signals were segmented into 1500‐ms epochs (500 ms prestimulation to 1000 ms poststimulation) time‐locked to the stimulation pulse. Data recorded from contacts of the whole shaft when stimulation was applied to one of the contact pairs, and contacts not located within the gray matter were discarded. We also excluded data recorded within an Euclidean distance of 10 mm from the stimulation site [12, 22]. Owing to variations in the shapes of evoked waves, we determined the root mean square (RMS) of the N1 (10–50 ms) and N2 (80–250 ms) periods to represent the early and late responses, respectively. Notably, these two components might indicate different connection types [12, 23]. Unlike structural connectivity, an asymmetric connection pattern may arise when the two pairs serve as stimulation and recording sites, respectively. Therefore, we explored effective connectivity in two directions: Inflow and outflow [22]. In our bipolar stimulation paradigm, stimulation was delivered between adjacent electrode contacts. To define the directionality, we adopted a stimulation‐centric framework: when a bipolar pair was stimulated, and responses were recorded at a distant site, the signal was classified as ‘outflow’ from the stimulated region to the recording region. Conversely, when stimulation occurred at a non‐SOZ site and responses were observed at the SOZ or resection zone, the signal was considered “inflow.” Herein, we primarily explored effective connectivity from two perspectives: (1) The connectivity between the SOZ and the surgically resected and nonresected regions, and (2) The connectivity between the resected and nonresected regions. The following parameters from SPES experiments were included in the final analysis (Figures 1 and 2). (1) IR‐N1/N2‐OUT/IN, which represent the N1/N2 RMS values of responses observed between the SOZ and other recording sites within the resected area. The clinical team determined the SOZ during the multidisciplinary seminar before resection surgery. Notably, “IR” stands for “in resection” and represents the connectivity between the SOZ and resection area. On the other hand, “OUT” represents the connectivity of the SOZ's outflow direction (stimulating the SOZ and observing responses at other sites within the resection). Finally, “IN” represents the connectivity of the SOZ's inflow direction (stimulating other sites and observing responses at SOZ). (2) OR‐N1/N2‐OUT/IN, where “OR” stands for “out of resection” and represents the connectivity between the SOZ and nonresected area. (3) IO‐N1/N2‐OUT/IN, where “IO” stands for “in and out of the resection” and reflects the connectivity between the resected and nonresected areas; “OUT” represents the connectivity of the outflow direction of the resection area (stimulating the sites within the resection area and observing responses at sites out of resection); and “IN” represents the connectivity of the inflow direction of the resection area (stimulating sites out of resection and observing responses at the resection area). While N1/N2 RMS amplitudes provide a robust and summary measure of evoked response strength, they do not capture detailed waveform morphology, such as onset latency or peak delay. In our study, only amplitude‐based features were analyzed. We acknowledge that the lack of latency and shape characteristics may limit the interpretability of connectivity directionality and response dynamics.

### 2.5 Statistical Analysis

In our study, the normality of continuous variables was assessed using the Shapiro–Wilk test. Normally distributed data that satisfied the homogeneity of variance assumption were compared using Student's t‐tests. On the other hand, non‐normally distributed data that did not meet the homogeneity of variance assumption were compared using nonparametric tests. Categorical data were compared using Chi‐square tests. False discovery rate (FDR) correction was used to address the risk of Type I error inflation from multiple univariate comparisons. Results with *p* < 0.05 were considered statistically significant. Model performance was assessed using various metrics, including accuracy, sensitivity, specificity, F1‐score, the receiver‐operating characteristic (ROC) curve, and area under the curve (AUC). Statistical analyses and model construction were performed using R software 4.3.0.

### 2.6 Classification of Surgical Outcomes

Based on clinical data and CCEP parameters, a feature vector was created for each patient. The features were first standardized using z‐scores and then partitioned into two groups in a 3:1 ratio: training and test sets. The training set was imported into the linear support vector machine (SVM) for binary classification between the SF and nSF groups. The mean value of the 5‐fold cross‐validation (CV) method was used to evaluate model performance and reliability. All model development steps, including 5‐fold cross‐validation and hyperparameter tuning, were conducted solely within the training set. The linear SVM classifier's hyperparameter C (penalty parameter) was optimized using grid search across a logarithmic scale (e.g., C = 0.01, 0.1, 1, 10, 100), with model performance evaluated via cross‐validation. The confusion matrix and ROC curve analyses were applied, and the accuracy, precision, specificity, and area under the ROC (AUROC) curve values were calculated. Feature importance was interpreted using SHAP (SHapley Additive exPlanations) values. In this study, SHAP analysis was implemented using the fastshap package in R software, which approximates Shapley values efficiently for any supervised learning model. This allowed us to compute feature contributions for each individual prediction and generate summary and force plots to visualize model interpretability [24]. A SHAP summary plot was generated to visualize the contributions of each feature to the model [25].

## 3 Results

### 3.1 Demographic Information

This study involved 56 TLE patients (30 females and 26 males) who underwent SEEG electrode implantation for epileptogenic zone localization followed by tailored ATL at our epilepsy center. Twenty‐eight had surgery on their left side. All patients were followed up for at least 1 year, with a mean follow‐up time of 18.43 ± 4.25 months. The patients were later categorized into two groups: Seizure‐free (SF; *N* = 30) and nonseizure‐free (nSF; *N* = 26). Table 1 presents the other details.

**TABLE 1:** Demographic information.

	ALL (*n* = 56)	SF (*n* = 30)	nSF (*n* = 26)	*p*
Sex, M/F	30/26	18/12	12/14	0.442
Age at onset	12.0 (0.5–30.0)	11.8 (0.5–26.0)	12.5 (1.0–30.0)	0.736
Age at surgery	24.0 (8.5–42.0)	24.5 (8.5–36.0)	24.0 (12.0–42.0)	0.329
Duration of epilepsy	10.0 (1.0–32.0)	9.5 (1.0–24.0)	10.5 (2.5–32.0)	0.598
Lateral, L/R	28/28	18/12	10/16	0.180
HS, Y/N	29/27	16/14	13/13	1.000
ASMs	3.0 ± 1.0	3.0 ± 1.0	3.5 ± 2.5	0.912
MRI, POS/NEG	32/24	19/11	13/13	0.462
GTCS, Y/N	29/27	11/19	18/8	0.030
Memory, Normal/Abnormal	20/36	13/17	7/19	0.318
Follow‐up time	18.43 ± 4.25	18.80 ± 4.24	18.00 ± 4.21	0.504
No. of electrodes	11.96 ± 2.00	12.27 ± 1.76	12.12 ± 1.89	0.397
Resection volume (cm^3^)	17.08 ± 4.19	17.65 ± 5.11	17.36 ± 4.66	0.518
Pathology (FCD I/FCD II/FCD IIIa/HS/NS)	12/12/18/9/5	4/7/12/4/3	8/5/6/5/2	0.443

Abbreviations: HS, hippocampal sclerosis; NS, nonspecific.

### 3.2 Comparison of CCEP Parameters

**FIGURE 3:**
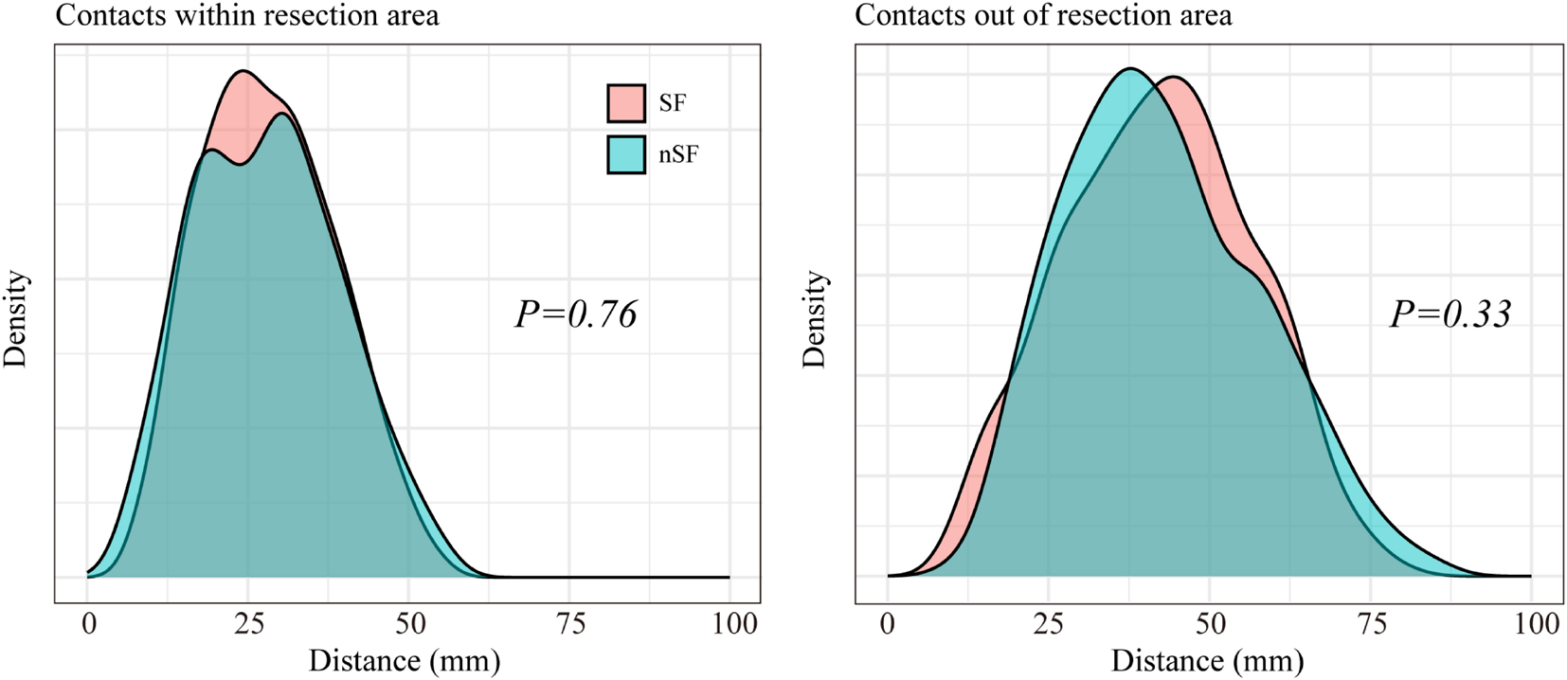
Distribution of SEEG contacts. (A) Distance distribution between contacts within the resection area and SOZ contacts; and (B) Distance distribution between contacts out of the resection area and SOZ contacts.

Notably, differences in electrode implantation distribution patterns between patients may cause bias in the results. Clinically, SEEG electrode implantation is often employed when preoperative noninvasive evaluations yield inconsistent results or when the resection area cannot be clearly defined. The electrode implantation plan often focuses on covering the hypothesized region while sticking to a specific pattern to avoid missing potential epileptogenic zones (for certain TLE types, the epileptogenic zone might extend beyond the temporal lobe [26]). Herein, we only included patients whose electrode implantation followed the “perisylvian” pattern, in which the electrodes primarily cover the medial and lateral temporal structures, orbito‐frontal cortex (OFC), anterior insula (AI), insulo‐opercular region (IOR), and anterior cingulate gyrus (ACG) [27]. This approach could help reduce the impact of different electrode distribution patterns between patients on the results. We further compared the distance between the SOZ contacts and other contacts in the SF and nSF groups, revealing no statistically significant difference in the distance both for contacts within and outside the resection area (Figure 3).

**FIGURE 4:**
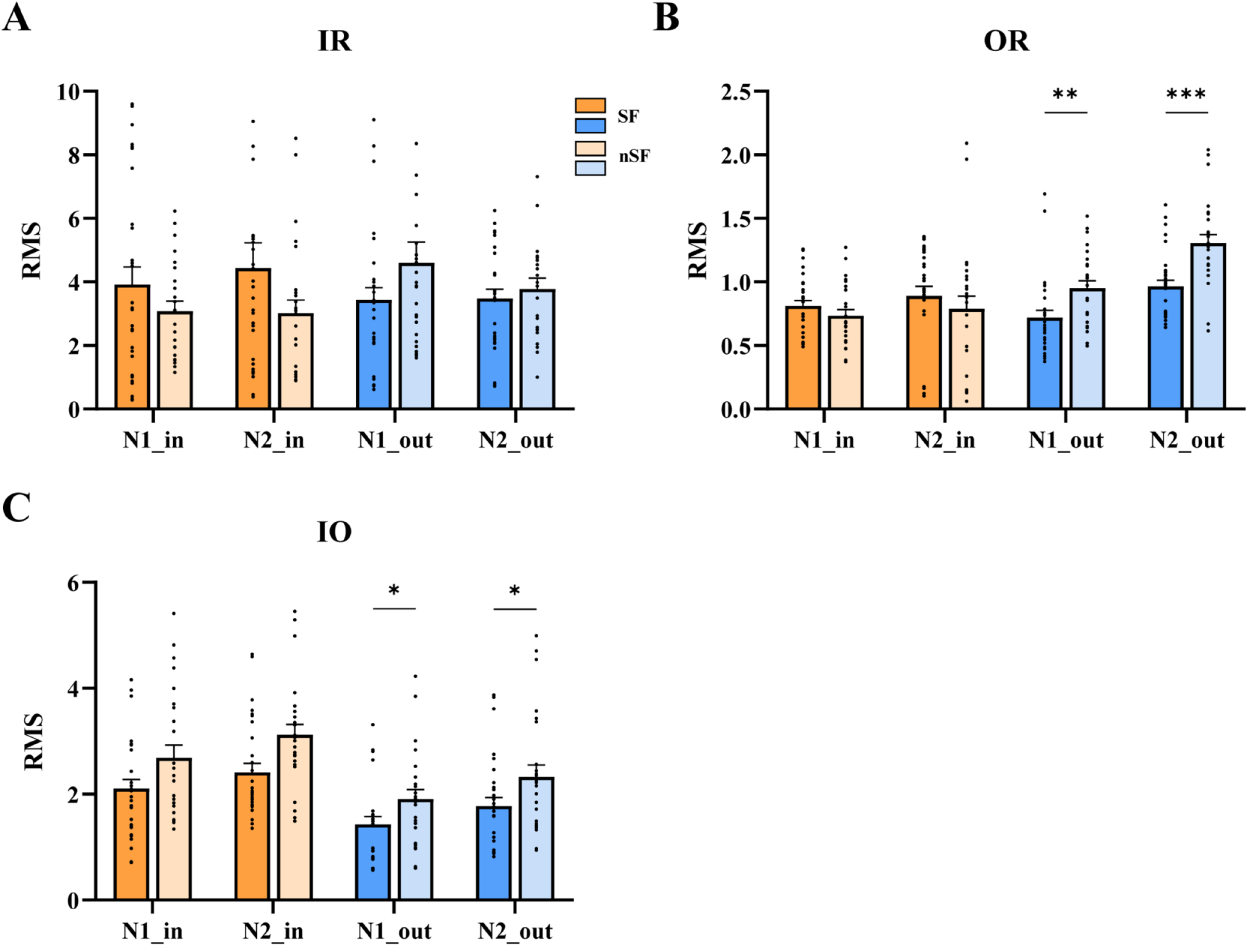
Comparison of CCEP parameters: (A) Comparison of the connectivity between SOZ contacts and other contacts within the resection area between the SF and nSF groups; (B) Comparison of the connectivity between SOZ contacts and other contacts out of the resection area between the SF and nSF groups; and (C) Comparison of the connectivity between contacts within the resection area and those out of the resection area between the SF and nSF groups. *: *p* < 0.05, **: *p* < 0.01, ***: *p* < 0.001.

We compared differences in CCEP parameters between different regions using univariate analysis. The SOZ electrodes and other electrodes within the resection area exhibited no significant difference between the SF and nSF groups. However, between SOZ contacts and contacts outside the resection area, both N1_out and N2_out differed between the two groups. Compared to the SF group [0.72 ± 0.30 (*p* = 0.006) and 0.96 ± 0.26 (*p* < 0.001)], the nSF group [0.95 ± 0.29 and 1.30 ± 0.34, respectively] showed higher N1_out and N2_out values. The two patient groups also showed differences in connections between the resection area and outside the resection area. Compared to the SF group [1.43 ± 0.78 (*p* = 0.041) and 1.77 ± 0.86 (*p* = 0.045)], the nSF group exhibited higher N1_out and N2_out values [1.90 ± 0.90 and 2.33 ± 1.11, respectively] (Figure 4).

### 3.3 Prediction Model Based on Clinical Information and CCEP Parameters

We constructed a prediction model based on 21 features: (1) There were nine clinical features (patients' age at onset and surgery, epilepsy duration, lateral of the surgery, with or without hippocampal sclerosis (HS), number of ASMs, positive or negative MRI, with or without GTCS, and presurgical memory condition). Categorical variables were transformed into dummy variables via one‐hot coding. (2) The remaining 12 features were CCEP parameters. The correlation matrix of these features revealed that no two features had a correlation coefficient > 0.8, indicating that the features captured a wide range of information.

**FIGURE 5:**
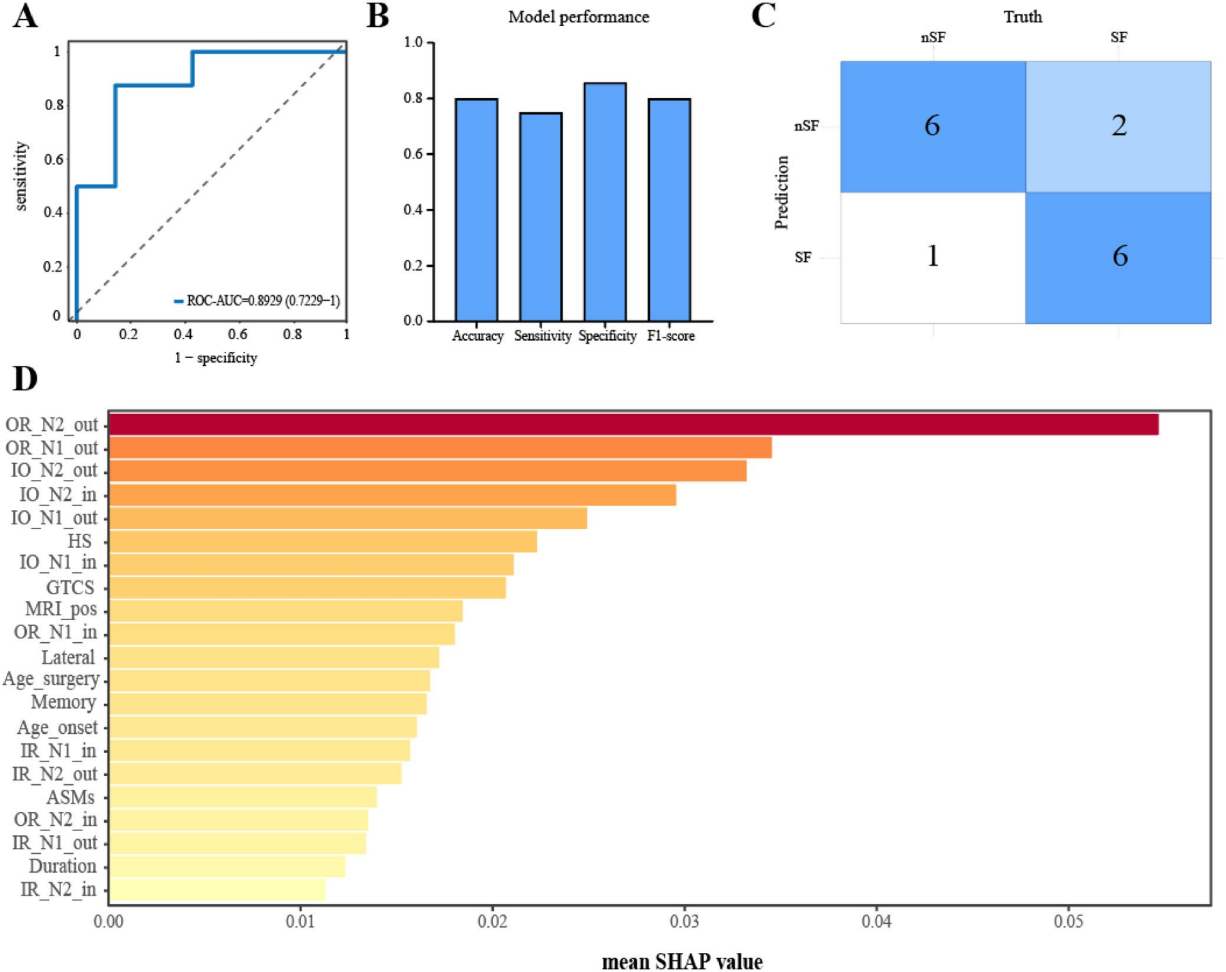
Machine learning (ML) model performance: (A) The model's ROC curve in the testing set; (B) Model performance evaluation metrics; (C) Confusion Matrix for the SVM model during cross‐validation; and (D) SHAP analysis‐derived feature importance.

**FIGURE 6:**
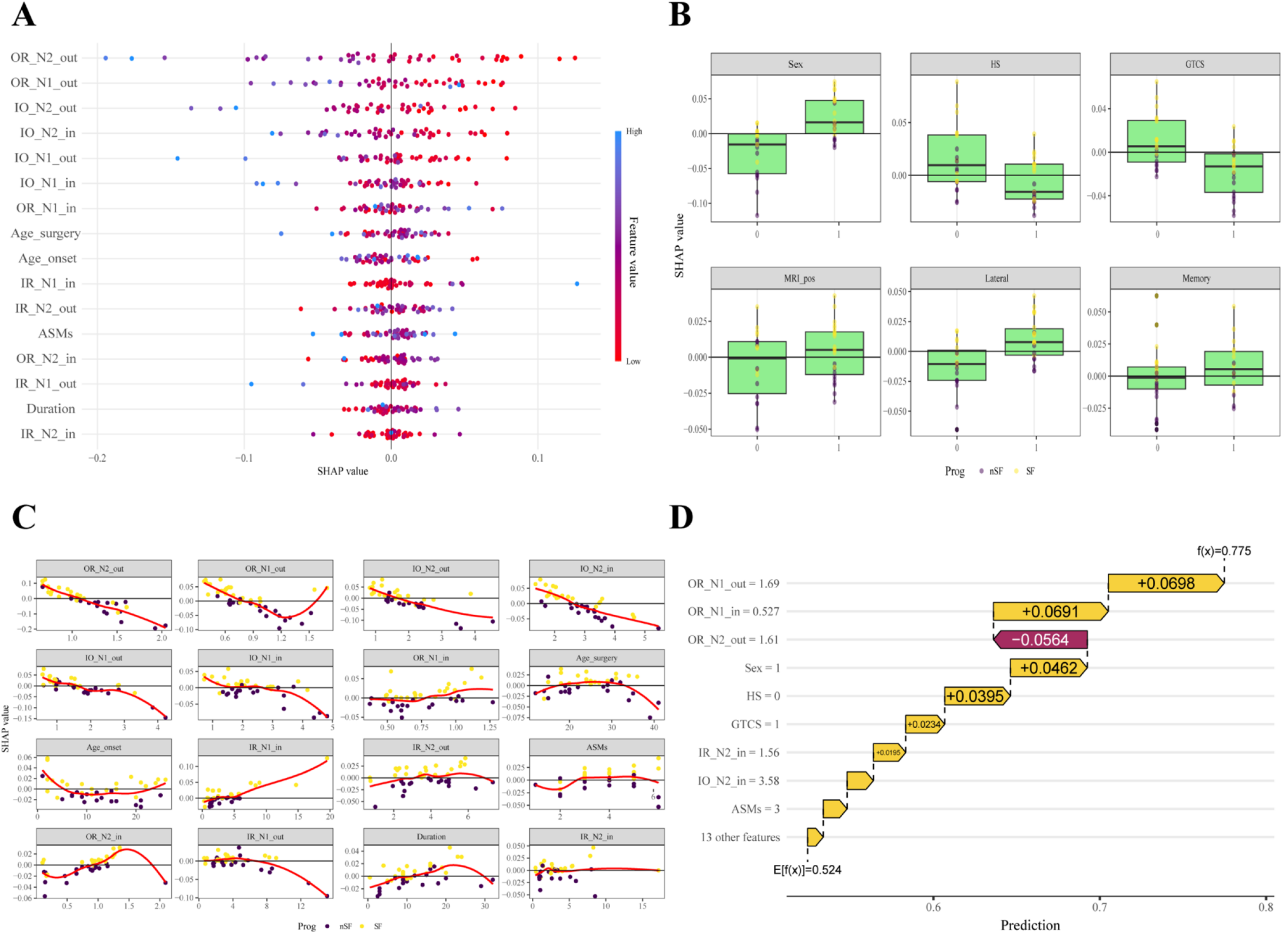
SHAP diagram of the SVM model: (A) Feature attributes of the model's continuous variables. Each line represents a feature, the x‐axis represents SHAP values (highlighting the impact of the feature on the outcome), and each point represents a sample; (B) SHAP value box plot for the model's categorical features; (C) SHAP value change trend diagram for the model's continuous variables; and (D) SHAP force plot for one sample.

The linear SVM model generated from these features through cross‐validation had accuracy, sensitivity, specificity, and F1‐score values of 0.800, 0.750, 0.857, and 0.800, respectively. Figure 5 shows the model's confusion matrix and ROC curves. The model's AUC‐ROC value was 0.893. We further performed SHAP analysis to assess the importance of each characteristic variable in the SVM model and specific contributions to model prediction, revealing that OR_N2_out and OR_N1_out were the two most important features. These values correlated positively with the likelihood of postoperative epilepsy recurrence. Moreover, the SHAP results confirmed that the connection strength within and outside the resection area was an important factor influencing surgical prognosis (Figure 6).

## 4 Discussion

Herein, we discovered that the effective connectivity strength obtained through the CCEP method can be used to predict TLE patients' surgical outcomes following tailored ATL. To the best of our knowledge, this is a pioneering study to construct a model that can directly predict patients' surgical outcomes based on effective connectivity relationships between resection areas, with seizure freedom as the gold standard after 1 year of resection. Furthermore, we found that the effective connectivity strength from the SOZ region to the area outside the resection zone was the most important factor in predicting surgical outcomes. Based on these insights, it is plausible that our predictive model could help predict surgical outcomes for TLE patients with implanted SEEG electrodes. Moreover, epilepsy surgeons could use CCEP‐obtained effective connectivity results to determine the appropriate surgical resection boundaries, thus improving surgical outcomes.

Among known epilepsies, TLE is the most common type of focal epilepsy and the most frequently treated with resective epilepsy surgery. It is well known that TLE could be considerably more complex, as it involves various temporal and extratemporal lobe networks contributing to the extent of the epileptogenic region and directly impacting seizure outcomes [28]. In this regard, it is noteworthy that neuroimaging techniques enabled the noninvasive study of pathologic structural and functional networks of epilepsy. For instance, quantitative MRI morphometric studies revealed coupled structural alterations in TLE patients, as well as consistent associations between TLE and bilateral neocortical atrophy, which could affect wide brain regions [29, 30]. Furthermore, diffusion‐weighted MRI analysis revealed that the surgical outcomes of TLE patients could be predicted by assessing the resection of key nodes in the network [31]. It was also reported that functional connectivity patterns from rs‐fMRI (resting state fMRI) successfully predicted surgical outcomes in TLE patients [32]. These findings highlight the potential significance of assessing surgical outcomes based on brain network characteristics. Compared to neuroimaging analysis, iEEG recording has a high temporal resolution and could illuminate epilepsy networks from a different perspective. Among the various EEG connectivity analysis methods, effective connectivity acquired from SPES experiments could provide directional connectivity results with causal information. The network patterns of effective connectivity were previously reported to correlate positively with epileptogenic networks, highlighting CCEP's potential in representing the epileptogenicity of brain regions [12, 16]. Multiple studies have attempted to leverage CCEP in the preoperative localization of the SOZ region and explore the epileptogenic network [19, 33, 34]. Nonetheless, these studies did not incorporate actual surgical resection boundaries and long‐term postoperative outcomes, limiting the clinical translation of their findings. They were largely descriptive and did not incorporate patient‐specific surgical resection boundaries into predictive frameworks. Additionally, most previous works focused on CCEP amplitude or latency features without integrating them into multivariate models with clinical outcome labels. In contrast, our study introduces a novel analytical pipeline that combines individualized resection zone masks, directional CCEP parameters, and clinical features into a machine learning‐based outcome prediction model, with interpretable feature attribution via SHAP and validation through cross‐validation and held‐out test sets. This modeling approach enables direct translational utility by informing surgical planning and outcome stratification at the individual level. According to the results of univariate tests, the connectivity from the SOZ to areas outside the resection, as well as the connectivity from the resected area to areas outside the resection, was higher in the nonseizure‐free group. We further constructed a predictive model for surgical outcomes incorporating CCEP parameters and clinical data. As earlier mentioned, we found that the effective connectivity strength from the SOZ region to the area outside the surgical resection zone was the most important factor influencing patients' long‐term prognosis. Furthermore, the early (N1) and late (N2) responses received outside the resection zone when stimulating the SOZ region both played important roles in the model. According to previous research, the N1‐evoked response reflects early pyramidal neuronal activation, while the N2 response represents a long‐lasting inhibition [35, 36]. Other studies that compared CCEP with other network analysis methods further revealed that the N1 of the CCEP partially reflected the structural and functional connectivity of the two sites, while the N2 may partially reflect functional connectivity [37, 38]. Strong N1/N2 outflow potentials from SOZ to resected regions could indicate preserved effective connectivity from pathological hubs to broader cortex, consistent with incomplete network disconnection in nSF patients. Conversely, lower outflow responses may signal successful removal of critical propagation pathways. Additionally, a previous DTI‐based study associated the presence of abnormal network nodes outside the surgical resection area with poorer surgical outcomes in TLE patients [31]. Moreover, Taylor et al. constructed a normative map of brain dynamics via iEEG band power analysis, revealing that poor surgical outcomes correlated with residual abnormal areas postsurgery, a phenomenon that was identified after comparison with the normative map [39]. Besides illuminating the crucial link between the complete resection of abnormal areas and postoperative seizure relief, these findings also highlight the significance and challenges of precise presurgical planning for resection areas in epilepsy surgery. Our results suggested that the resection of regions closely connected with the SOZ could affect patients' surgical outcomes. Although these areas may be part of the same epileptic network as the SOZ, they often lie beyond the regions identified via neuroimaging or presurgical evaluation, causing incomplete resection during surgery and ultimately affecting patients' prognosis. The connectivity between the resection area and regions outside the resection area also emerged as an important factor influencing surgical outcomes. SHAP analysis results revealed that the values of IO_N2_IN and IO_N2_OUT correlated positively with the likelihood of seizure recurrence postsurgery. The study by Shah et al. found that connectivity within the resection zone is associated with good postsurgical outcomes using functional connectivity analysis methods [40]. This differs from our findings, which may be due to our study only calculating the connectivity between the SOZ and other contacts within the resection zone. Future studies incorporating more connectivity characteristics are needed. Sinha et al. developed dynamical network models for predicting surgical outcomes using the iEEG connectivity matrix. Notably, this patient‐specific model could also suggest alternative resection sites in patients with recurrent seizures. This study, along with ours, further highlighted the potential significance of predicting surgical outcomes based on network connectivity characteristics [41]. Furthermore, we also used SHAP analysis to improve the model's interpretability, crucially enhancing the potential for further clinical application and promotion. Specifically, improving the model's interpretability could help users better understand how the model works.

Despite its valuable insights, this study also had some limitations. First, its sample size was relatively small. However, few studies have integrated the surgical resection area and effective connectivity for clinical outcome prediction. Therefore, this study offers reliable theoretical support for future larger sample sizes, multicenter studies. Second, owing to challenges in iEEG acquisition and standardizing CCEP testing protocols, this was a single‐center study, and no external validation set was used to assess the model's performance. However, we used five‐fold cross‐validation to ensure the reliability of the model's performance evaluation results. Third, the participants included herein were all TLE patients, highlighting the need to validate the findings further in patients with more diverse pathological types. In the future, it will be necessary to validate our findings in independent cohorts from multiple epilepsy centers and to assess the applicability of the model to other epilepsy types and pathologies, such as frontal lobe epilepsy or nonlesional cases. These steps will be critical for evaluating the generalizability and clinical utility of the proposed framework.

## 5 Conclusion

Herein, we predicted epilepsy surgery outcomes using effective connectivity analysis, representing an important addition to the realm of predicting surgical prognosis based on brain network characteristics. The connectivity between different surgical areas was an important factor influencing surgical outcomes. Moreover, we constructed an interpretable model with convenient clinical applicability. Besides allowing for the prediction of the patient's surgical prognosis via surgical resection area preplanning, this model could be further used to adjust the surgical plan presurgery, increasing the likelihood of patients being seizure‐free postoperatively. While promising, the potential use of this model to guide resection strategies remains hypothetical and warrants future prospective studies.

## Disclosure

The authors have nothing to report.

## Conflicts of Interest

The authors declare no conflicts of interest.

## Supporting information


**Table S1:** The proportion of data excluded.
**Table S2:** The Dice Coefficient of two raters.
**Table S3:** Comparison of CCEP parameters between different pathological subtypes and the overall cohort.
**Table S4:** Distribution of SEEG electrode contacts in all patients.

## Data Availability

The data that support the findings of this study are available on request from the corresponding author. The data are not publicly available due to privacy or ethical restrictions.
