# Supplementary material for: Effective Connectivity Predicts Surgical Outcomes in Temporal Lobe Epilepsy: A SEEG Study
Source: CNS Neurosci Ther. 2025 Aug 26;31(8):e70563. doi: 10.1111/cns.70563 (PMC12378694; doi:10.1111/cns.70563)
Supplement: Supplementary file 1 — Table S1: The proportion of data excluded. Table S2: The Dice Coefficient of two raters. Table S3: Comparison of CCEP parameters between different pathological subtypes and the overall cohort. Table S4: Distribution of SEEG electrode contacts in all patients. [file CNS-31-e70563-s001.docx]

Table S1 The proportion of data excluded

| Patients | Number of electrodes | Number of contacts | Number of discarded contacts | Proportation of discarded contacts (%) |
| --- | --- | --- | --- | --- |
| 1 | 14 | 206 | 29 | 14.08 |
| 2 | 13 | 207 | 26 | 12.56 |
| 3 | 10 | 155 | 35 | 22.58 |
| 4 | 9 | 137 | 37 | 27.01 |
| 5 | 10 | 143 | 33 | 23.08 |
| 6 | 9 | 129 | 30 | 23.26 |
| 7 | 14 | 198 | 35 | 17.68 |
| 8 | 12 | 189 | 27 | 14.29 |
| 9 | 12 | 182 | 29 | 15.93 |
| 10 | 9 | 139 | 32 | 23.02 |
| 11 | 13 | 183 | 25 | 13.66 |
| 12 | 13 | 207 | 29 | 14.01 |
| 13 | 11 | 172 | 33 | 19.19 |
| 14 | 14 | 202 | 33 | 16.34 |
| 15 | 13 | 187 | 30 | 16.04 |
| 16 | 12 | 172 | 27 | 15.7 |
| 17 | 15 | 219 | 26 | 11.87 |
| 18 | 10 | 150 | 30 | 20 |
| 19 | 12 | 178 | 30 | 16.85 |
| 20 | 15 | 219 | 25 | 11.42 |
| 21 | 13 | 198 | 28 | 14.14 |
| 22 | 10 | 143 | 27 | 18.88 |
| 23 | 14 | 204 | 24 | 11.76 |
| 24 | 12 | 177 | 30 | 16.95 |
| 25 | 13 | 194 | 34 | 17.53 |
| 26 | 12 | 187 | 36 | 19.25 |
| 27 | 13 | 187 | 28 | 14.97 |
| 28 | 10 | 150 | 24 | 16 |
| 29 | 14 | 213 | 34 | 15.96 |
| 30 | 11 | 155 | 27 | 17.42 |
| 31 | 11 | 167 | 23 | 13.77 |
| 32 | 12 | 172 | 34 | 19.77 |
| 33 | 13 | 184 | 32 | 17.39 |
| 34 | 12 | 191 | 28 | 14.66 |
| 35 | 15 | 239 | 35 | 14.64 |
| 36 | 15 | 234 | 34 | 14.53 |
| 37 | 10 | 146 | 31 | 21.23 |
| 38 | 12 | 170 | 23 | 13.53 |
| 39 | 10 | 154 | 33 | 21.43 |
| 40 | 12 | 179 | 33 | 18.44 |
| 41 | 9 | 128 | 37 | 28.91 |
| 42 | 10 | 150 | 32 | 21.33 |
| 43 | 9 | 127 | 34 | 26.77 |
| 44 | 10 | 158 | 34 | 21.52 |
| 45 | 13 | 189 | 37 | 19.58 |
| 46 | 9 | 138 | 36 | 26.09 |
| 47 | 9 | 132 | 36 | 27.27 |
| 48 | 9 | 135 | 37 | 27.41 |
| 49 | 15 | 226 | 36 | 15.93 |
| 50 | 13 | 187 | 25 | 13.37 |
| 51 | 14 | 223 | 34 | 15.25 |
| 52 | 14 | 218 | 29 | 13.3 |
| 53 | 9 | 143 | 26 | 18.18 |
| 54 | 13 | 205 | 31 | 15.12 |
| 55 | 15 | 228 | 25 | 10.96 |
| 56 | 15 | 238 | 27 | 11.34 |

Table S2 The Dice Coefficient of two raters

| Patients | Rater 1 Volume (cm^3^) | Rater 2 Volume (cm^3^) | Dice Coefficient |
| --- | --- | --- | --- |
| 1 | 13.75 | 13.06 | 0.79 |
| 2 | 19.51 | 23.02 | 0.94 |
| 3 | 17.32 | 18.88 | 0.88 |
| 4 | 15.99 | 16.63 | 0.85 |
| 5 | 11.56 | 9.94 | 0.74 |
| 6 | 11.56 | 9.94 | 0.74 |
| 7 | 10.58 | 8.68 | 0.71 |
| 8 | 18.66 | 21.46 | 0.92 |
| 9 | 16.01 | 16.65 | 0.85 |
| 10 | 17.08 | 18.45 | 0.88 |
| 11 | 10.21 | 8.27 | 0.71 |
| 12 | 19.7 | 23.44 | 0.94 |
| 13 | 18.32 | 20.7 | 0.91 |
| 14 | 12.12 | 10.67 | 0.75 |
| 15 | 11.82 | 10.28 | 0.75 |
| 16 | 11.83 | 10.29 | 0.75 |
| 17 | 13.04 | 12 | 0.78 |
| 18 | 15.25 | 15.4 | 0.83 |
| 19 | 14.32 | 13.89 | 0.81 |
| 20 | 12.91 | 11.88 | 0.77 |
| 21 | 16.12 | 16.76 | 0.85 |
| 22 | 11.39 | 9.8 | 0.73 |
| 23 | 12.92 | 11.89 | 0.77 |
| 24 | 13.66 | 12.98 | 0.79 |
| 25 | 14.56 | 14.27 | 0.81 |
| 26 | 17.85 | 19.81 | 0.9 |
| 27 | 12 | 10.56 | 0.75 |
| 28 | 15.14 | 15.29 | 0.83 |
| 29 | 15.92 | 16.56 | 0.85 |
| 30 | 10.46 | 8.58 | 0.71 |
| 31 | 16.08 | 16.72 | 0.85 |
| 32 | 11.71 | 10.19 | 0.74 |
| 33 | 10.65 | 8.84 | 0.72 |
| 34 | 19.49 | 23 | 0.94 |
| 35 | 19.66 | 23.4 | 0.94 |
| 36 | 18.08 | 20.25 | 0.9 |
| 37 | 13.05 | 12.01 | 0.78 |
| 38 | 10.98 | 9.22 | 0.72 |
| 39 | 16.84 | 18.02 | 0.87 |
| 40 | 14.4 | 14.11 | 0.81 |
| 41 | 11.22 | 9.54 | 0.73 |
| 42 | 14.95 | 14.95 | 0.82 |
| 43 | 10.34 | 8.38 | 0.71 |
| 44 | 19.09 | 22.14 | 0.93 |
| 45 | 12.59 | 11.33 | 0.76 |
| 46 | 16.63 | 17.79 | 0.87 |
| 47 | 13.12 | 12.07 | 0.78 |
| 48 | 15.2 | 15.35 | 0.83 |
| 49 | 15.47 | 15.78 | 0.84 |
| 50 | 11.85 | 10.31 | 0.75 |
| 51 | 19.7 | 23.44 | 0.94 |
| 52 | 17.75 | 19.7 | 0.89 |
| 53 | 19.39 | 22.88 | 0.93 |
| 54 | 18.95 | 21.98 | 0.92 |
| 55 | 15.98 | 16.62 | 0.85 |
| 56 | 19.22 | 22.49 | 0.93 |

Table S3 Comparison of CCEP parameters between different pathological subtypes and the overall cohort

|  | FCD I | P | FCD II | P | FCD IIIa | P | HS | P | NS | P |
| --- | --- | --- | --- | --- | --- | --- | --- | --- | --- | --- |
| IR-N1-OUT | 3.32±3.72 | 0.44 | 5.38±3.44 | 0.06 | 3.38±3.11 | 0.34 | 4.05±3.69 | 0.19 | 4.86±3.74 | 0.73 |
| IR-N2-OUT | 3.95±1.49 | 0.51 | 4.04±1.58 | 0.41 | 3.84±1.01 | 0.49 | 4.40±1.92 | 0.27 | 2.96±0.85 | 0.18 |
| IR-N1-IN | 5.31±3.11 | 0.26 | 1.59±4.18 | 0.08 | 3.07±3.87 | 0.36 | 2.62±3.74 | 0.31 | 5.33±3.28 | 0.45 |
| IR-N2-IN | 3.18±2.26 | 0.46 | 3.62±3.59 | 0.89 | 3.27±3.17 | 0.57 | 3.16±1.58 | 0.39 | 4.30±2.74 | 0.71 |
| OR-N1-OUT | 0.72±0.30 | 0.3 | 0.73±0.27 | 0.28 | 0.97±0.28 | 0.08 | 0.86±0.20 | 0.71 | 0.78±0.45 | 0.83 |
| OR-N2-OUT | 1.20±0.44 | 0.57 | 0.93±0.39 | 0.13 | 1.09±0.36 | 0.71 | 1.20±0.26 | 0.44 | 1.30±0.36 | 0.34 |
| OR-N1-IN | 0.69±0.22 | 0.24 | 0.76±0.24 | 0.79 | 0.71±0.25 | 0.37 | 0.81±0.45 | 0.84 | 0.64±0.07 | 0.06 |
| OR-N2-IN | 0.88±0.52 | 0.81 | 0.69±0.51 | 0.36 | 0.84±0.41 | 1 | 1.00±0.41 | 0.32 | 0.69±0.41 | 0.47 |
| IO-N1-OUT | 1.32±0.61 | 0.13 | 1.63±0.78 | 0.91 | 1.67±1.09 | 0.96 | 2.10±0.84 | 0.17 | 1.07±0.67 | 0.12 |
| IO-N2-OUT | 1.48±1.27 | 0.18 | 2.22±1.10 | 0.59 | 1.55±0.93 | 0.07 | 2.02±1.03 | 0.97 | 1.86±1.54 | 0.81 |
| IO-N1-IN | 1.88±1.06 | 0.16 | 2.80±0.82 | 0.14 | 2.14±0.88 | 0.35 | 2.28±1.00 | 0.79 | 2.88±0.67 | 0.18 |
| IO-N2-IN | 2.77±0.66 | 0.92 | 2.70±1.05 | 0.9 | 2.70±0.93 | 0.86 | 2.70±0.87 | 0.91 | 2.84±1.26 | 0.87 |

Table S4 Distribution of SEEG electrode contacts in all patients

| Patients | HIP | PHG | AMYG | STG | MTG | ITG | TPO | PreCG | SFG | MFG | IFG | SMA | OLF | REC | OFC | INS | ACC | LING | FFG |
| --- | --- | --- | --- | --- | --- | --- | --- | --- | --- | --- | --- | --- | --- | --- | --- | --- | --- | --- | --- |
| 1 | 5 | 27 | 4 | 24 | 21 | 15 | 6 | 6 | 16 | 22 | 11 | 1 | 2 | 3 | 1 | 11 | 2 | 0 | 0 |
| 2 | 4 | 25 | 4 | 19 | 18 | 22 | 6 | 6 | 13 | 19 | 10 | 8 | 6 | 3 | 3 | 12 | 3 | 0 | 0 |
| 3 | 8 | 17 | 4 | 15 | 14 | 17 | 1 | 3 | 18 | 4 | 3 | 1 | 2 | 0 | 2 | 7 | 4 | 0 | 0 |
| 4 | 7 | 15 | 4 | 19 | 10 | 9 | 0 | 0 | 5 | 6 | 7 | 0 | 1 | 1 | 0 | 10 | 6 | 0 | 0 |
| 5 | 3 | 17 | 4 | 23 | 19 | 15 | 1 | 6 | 3 | 4 | 5 | 0 | 1 | 0 | 1 | 6 | 2 | 0 | 0 |
| 6 | 4 | 19 | 5 | 15 | 13 | 10 | 3 | 0 | 2 | 5 | 4 | 0 | 4 | 4 | 2 | 5 | 4 | 0 | 0 |
| 7 | 7 | 19 | 4 | 19 | 21 | 14 | 0 | 6 | 15 | 21 | 13 | 7 | 3 | 0 | 1 | 9 | 4 | 0 | 0 |
| 8 | 5 | 11 | 4 | 14 | 22 | 16 | 3 | 6 | 15 | 21 | 16 | 5 | 6 | 1 | 5 | 10 | 2 | 0 | 0 |
| 9 | 6 | 20 | 4 | 15 | 16 | 13 | 1 | 2 | 15 | 16 | 15 | 6 | 4 | 1 | 1 | 16 | 2 | 0 | 0 |
| 10 | 7 | 15 | 4 | 15 | 19 | 10 | 1 | 1 | 1 | 3 | 5 | 6 | 4 | 4 | 0 | 8 | 4 | 0 | 0 |
| 11 | 8 | 16 | 4 | 25 | 10 | 19 | 6 | 1 | 14 | 15 | 14 | 5 | 4 | 0 | 0 | 13 | 4 | 0 | 0 |
| 12 | 3 | 23 | 2 | 23 | 21 | 16 | 6 | 5 | 15 | 20 | 18 | 1 | 6 | 1 | 4 | 14 | 0 | 0 | 0 |
| 13 | 4 | 19 | 2 | 20 | 11 | 10 | 2 | 0 | 17 | 17 | 4 | 8 | 0 | 4 | 3 | 13 | 5 | 0 | 0 |
| 14 | 7 | 16 | 3 | 21 | 18 | 17 | 6 | 2 | 12 | 24 | 15 | 6 | 0 | 2 | 2 | 12 | 6 | 0 | 0 |
| 15 | 4 | 12 | 2 | 24 | 24 | 11 | 0 | 5 | 13 | 18 | 15 | 8 | 4 | 3 | 2 | 8 | 4 | 0 | 0 |
| 16 | 6 | 16 | 3 | 23 | 19 | 14 | 3 | 1 | 14 | 15 | 13 | 5 | 0 | 0 | 2 | 10 | 1 | 0 | 0 |
| 17 | 7 | 18 | 3 | 22 | 22 | 13 | 4 | 4 | 18 | 22 | 14 | 10 | 0 | 2 | 0 | 18 | 5 | 7 | 4 |
| 18 | 5 | 12 | 4 | 14 | 11 | 12 | 4 | 4 | 6 | 8 | 5 | 8 | 1 | 2 | 1 | 13 | 0 | 5 | 5 |
| 19 | 5 | 11 | 3 | 20 | 21 | 16 | 2 | 4 | 12 | 16 | 13 | 7 | 4 | 3 | 4 | 4 | 3 | 0 | 0 |
| 20 | 4 | 14 | 4 | 28 | 12 | 15 | 3 | 5 | 36 | 18 | 15 | 4 | 4 | 4 | 6 | 16 | 6 | 0 | 0 |
| 21 | 5 | 17 | 4 | 18 | 18 | 16 | 6 | 6 | 17 | 23 | 15 | 4 | 5 | 1 | 1 | 14 | 0 | 0 | 0 |
| 22 | 10 | 18 | 3 | 17 | 16 | 16 | 6 | 1 | 3 | 5 | 4 | 1 | 2 | 1 | 2 | 6 | 5 | 0 | 0 |
| 23 | 3 | 20 | 2 | 24 | 16 | 22 | 6 | 6 | 15 | 21 | 10 | 8 | 5 | 4 | 1 | 15 | 2 | 0 | 0 |
| 24 | 3 | 20 | 3 | 20 | 17 | 17 | 3 | 6 | 12 | 18 | 13 | 1 | 2 | 2 | 3 | 5 | 2 | 0 | 0 |
| 25 | 6 | 12 | 3 | 23 | 24 | 14 | 3 | 2 | 18 | 20 | 10 | 2 | 3 | 1 | 3 | 16 | 0 | 0 | 0 |
| 26 | 7 | 19 | 4 | 22 | 17 | 11 | 2 | 3 | 13 | 16 | 9 | 6 | 6 | 2 | 1 | 7 | 6 | 0 | 0 |
| 27 | 6 | 13 | 3 | 25 | 22 | 12 | 4 | 1 | 13 | 14 | 16 | 4 | 2 | 3 | 0 | 15 | 6 | 0 | 0 |
| 28 | 5 | 20 | 2 | 22 | 18 | 16 | 0 | 0 | 11 | 8 | 5 | 1 | 4 | 2 | 1 | 10 | 1 | 0 | 0 |
| 29 | 7 | 21 | 4 | 22 | 16 | 23 | 4 | 3 | 16 | 19 | 16 | 2 | 6 | 4 | 1 | 10 | 5 | 0 | 0 |
| 30 | 3 | 18 | 4 | 19 | 21 | 12 | 0 | 5 | 13 | 8 | 5 | 0 | 0 | 0 | 1 | 16 | 3 | 0 | 0 |
| 31 | 6 | 12 | 2 | 16 | 22 | 19 | 0 | 3 | 14 | 17 | 9 | 5 | 6 | 0 | 2 | 7 | 4 | 0 | 0 |
| 32 | 6 | 13 | 2 | 20 | 18 | 19 | 6 | 2 | 12 | 11 | 7 | 7 | 4 | 1 | 1 | 8 | 1 | 0 | 0 |
| 33 | 8 | 17 | 2 | 18 | 17 | 19 | 6 | 3 | 14 | 17 | 14 | 7 | 0 | 0 | 1 | 4 | 5 | 0 | 0 |
| 34 | 6 | 19 | 4 | 20 | 21 | 15 | 0 | 6 | 7 | 18 | 12 | 7 | 6 | 1 | 5 | 15 | 1 | 0 | 0 |
| 35 | 6 | 16 | 4 | 32 | 36 | 8 | 8 | 1 | 13 | 7 | 17 | 13 | 4 | 4 | 3 | 16 | 5 | 6 | 5 |
| 36 | 7 | 16 | 4 | 21 | 15 | 25 | 6 | 4 | 32 | 23 | 18 | 5 | 5 | 2 | 1 | 15 | 1 | 0 | 0 |
| 37 | 4 | 19 | 3 | 15 | 14 | 15 | 3 | 0 | 6 | 6 | 3 | 4 | 0 | 3 | 2 | 5 | 4 | 0 | 0 |
| 38 | 5 | 20 | 3 | 24 | 14 | 13 | 5 | 1 | 13 | 14 | 13 | 3 | 4 | 1 | 4 | 10 | 0 | 0 | 0 |
| 39 | 7 | 11 | 2 | 18 | 11 | 15 | 4 | 2 | 20 | 5 | 3 | 3 | 2 | 2 | 1 | 12 | 3 | 0 | 0 |
| 40 | 3 | 18 | 4 | 15 | 17 | 13 | 3 | 6 | 12 | 18 | 8 | 8 | 0 | 2 | 4 | 11 | 4 | 0 | 0 |
| 41 | 3 | 14 | 4 | 21 | 14 | 9 | 0 | 0 | 2 | 3 | 3 | 3 | 5 | 0 | 0 | 5 | 5 | 0 | 0 |
| 42 | 5 | 11 | 2 | 21 | 23 | 15 | 6 | 2 | 0 | 5 | 7 | 5 | 0 | 3 | 2 | 9 | 2 | 0 | 0 |
| 43 | 5 | 13 | 2 | 17 | 11 | 7 | 1 | 0 | 2 | 8 | 4 | 1 | 0 | 2 | 0 | 16 | 4 | 0 | 0 |
| 44 | 7 | 13 | 3 | 19 | 15 | 16 | 5 | 4 | 17 | 3 | 4 | 1 | 4 | 2 | 1 | 7 | 3 | 0 | 0 |
| 45 | 5 | 11 | 4 | 18 | 21 | 15 | 3 | 5 | 14 | 19 | 11 | 8 | 1 | 0 | 2 | 9 | 6 | 0 | 0 |
| 46 | 3 | 16 | 2 | 14 | 13 | 11 | 2 | 5 | 3 | 7 | 5 | 5 | 2 | 2 | 2 | 5 | 5 | 0 | 0 |
| 47 | 3 | 14 | 2 | 16 | 17 | 10 | 6 | 5 | 3 | 6 | 7 | 1 | 1 | 0 | 0 | 5 | 0 | 0 | 0 |
| 48 | 5 | 12 | 4 | 17 | 11 | 8 | 3 | 1 | 3 | 3 | 7 | 0 | 3 | 1 | 3 | 15 | 2 | 0 | 0 |
| 49 | 8 | 21 | 4 | 31 | 18 | 22 | 3 | 3 | 10 | 13 | 16 | 6 | 6 | 1 | 0 | 9 | 4 | 8 | 7 |
| 50 | 6 | 10 | 4 | 15 | 17 | 14 | 6 | 6 | 19 | 25 | 14 | 3 | 5 | 0 | 1 | 11 | 6 | 0 | 0 |
| 51 | 5 | 18 | 3 | 23 | 26 | 10 | 5 | 6 | 23 | 29 | 10 | 8 | 4 | 1 | 0 | 13 | 5 | 0 | 0 |
| 52 | 8 | 18 | 4 | 26 | 25 | 14 | 5 | 5 | 15 | 22 | 13 | 9 | 0 | 2 | 2 | 16 | 0 | 5 | 0 |
| 53 | 3 | 11 | 3 | 16 | 14 | 19 | 1 | 2 | 7 | 7 | 5 | 0 | 5 | 1 | 2 | 10 | 5 | 0 | 0 |
| 54 | 8 | 24 | 4 | 19 | 23 | 18 | 3 | 7 | 11 | 18 | 13 | 2 | 0 | 5 | 1 | 11 | 3 | 4 | 0 |
| 55 | 8 | 24 | 3 | 34 | 28 | 16 | 1 | 3 | 15 | 16 | 15 | 8 | 6 | 3 | 5 | 12 | 6 | 0 | 0 |
| 56 | 4 | 26 | 4 | 27 | 35 | 25 | 6 | 5 | 16 | 6 | 4 | 8 | 2 | 3 | 4 | 15 | 4 | 10 | 7 |

PreCG(Precentral gyrus), SFG(Superior frontal gyrus), MFG(Middle frontal gyrus), IFG(Inferior frontal gyrus), SMA(Supplementary motor area), OLF(Olfactory cortex), REC(Gyrus rectus), OFC(orbital gyrus), INS(Insula), ACC(Anterior cingulate cortex), MCC(middle cingulate cortex), PCC(posterior cingulate cortex),

HIP(Hippocampus), PHG(Parahippocampal gyrus), AMYG(Amygdala), STG(superior temporal gyrus), MTG(middle temporal gyrus), ITG(Inferior temporal gyrus), TPO(Temporal pole), LING(Lingual gyrus),FFG(Fusiform gyrus).
